# Beyond Taste and Nutrition Food Dimensions Addressed in Dietetic Consultations With Malnourished Home‐Dwelling Older Adults: A Qualitative Study

**DOI:** 10.1111/jhn.70290

**Published:** 2026-06-07

**Authors:** Matthijs Fleurke, Laura Bouwman, Jacqueline Langius, Spencer Moore

**Affiliations:** ^1^ Health and Society Group Wageningen University & Research Wageningen the Netherlands; ^2^ Nutrition & Dietetics The Hague University of Applied Sciences The Hague the Netherlands

**Keywords:** food dimensions, habitus, home‐dwelling older adults, malnutrition, qualitative research

## Abstract

**Introduction:**

The management of malnutrition in home‐dwelling older adults is challenging since they often must adjust long‐standing food habits developed throughout a lifetime. Dietitians aim to support these adjustments by applying patient‐centered care (PCC). Whether and how PCC‐based dietary advice fits patients' everyday handling of food remains, however, relatively unexplored. Specifically, it is unclear to what extent everyday dimensions of food, such as health, social, and cultural dimensions emerge during consultations, and who tends to raise them. This study aims to analyse and compare which food dimensions are raised by dietitians and patients during consultations and how these dimensions are embedded in patients' everyday lives.

**Methods:**

From November 2022 to March 2023, observations were conducted of 31 home visit consultations with home‐dwelling older adults by 8 dietitians in the Netherlands and 31 informal interviews with dietitians to provide further context about the visit. The participants included dietitians visiting malnourished, home‐dwelling older adults, the malnourished older adults themselves, and, if applicable, their informal caregivers. Bourdieu's sociological concept of habitus, a set of ingrained social structures that shape everyday thinking and acting, was applied to categorize food dimensions and to analyse how they are embedded in patients' everyday lives.

**Results:**

While dietitians customize their advice for each patient, they emphasize different dimensions of food. Dietitians primarily focus on ‘measuring and knowing’ and the ‘effect of diet therapy’, reflecting a rational and functional approach to food and malnutrition treatment. Patients emphasize social, emotional and temporal dimensions, bringing in a broader and personal approach to food and malnutrition.

**Conclusion:**

Dietitians emphasize rational and functional dimensions of food, while patients foreground social, emotional, and temporal dimensions. From a Bourdieusian perspective, dietitians' advice often reflects pragmatic and individualistic orientations. Recognizing these orientations can help dietitians further integrate the historical and social embeddedness of food, allowing their advice to better resonate with patients' habitus and thus with their everyday food‐related practices, attitudes, and behaviors.

## Summary

- Being aware of, and accounting for, the different dimensions of food can help dietitians balance their conversations with older adults and move beyond a narrowly functional focus.
- Recognising that older adults' food choices are shaped by social, cultural, and lifelong experiences helps dietitians tailor advice that fits more naturally into patients' everyday lives.

## 1 Introduction

Nutritional care provided by dietitians plays an important role in the management of malnutrition among home‐dwelling older adults [1, 2]. A key element of nutritional care is advice on how to increase or maintain nutritional intake. To facilitate necessary changes in patients' eating habits, dietitians may adopt an individualized approach, responsive to each patient's preferences, needs, values [3, 4, 5] and personal situation [6]. This approach has been labeled ‘patient centered care’ (PCC). PCC enhances patient satisfaction with nutrition care and improves dietary behaviors [5, 7, 8]. PCC is a broad concept and its application in dietetic consultations has been studied with regards to dietitian‐patient communication, interdisciplinary coordination and collaboration, and addressing the patients' primary medical concern [8].

An underexplored aspect of applying PCC in dietetics is how dietitians embed their advice into the patient's individual's life when it comes to dimensions of food that emerge during consultations. We define food dimensions as the diverse ways in which food integrates consciously or unconsciously into a person's life, whether practically, mentally, or socially. Food always has multiple dimensions in people's life [9, 10, 11]. For example, food may carry a health‐related dimension when a person associates it with concerns such as malnutrition. At the same time, it may involve a financial dimension when that person feels limited to choosing more affordable options [12]. Food dimensions range from concrete and experiential, like the dimension of taste, to abstract and theoretical, like the dimension of (specific) nutrient values. Furthermore, the importance of various food dimensions can vary among individuals, and their significance may evolve over time. The concept of the ‘multidimensionality of food’ introduces an everyday‐life perspective into dietetics by revealing that, due to its many dimensions, food is deeply embedded in people's everyday lives.

The literature used in dietetic education programs and nutrition research predominantly emphasizes physical‐nutritional dimensions of food, while giving less attention to dimensions related to the broader socio‐cultural meaning and role of food in everyday life [13]. This presents an opportunity to improve PCC by better incorporating the multidimensional nature of everyday food handling into dietetic consultations [7, 13, 14, 15], potentially improving their effectiveness [3]. This study aims to indicate which food dimensions tend to be raised by dietitians and patients during consultations and how these dimensions are embedded in patients' everyday lives.

### 1.1 Theoretical Background: Bourdieu's Concept of Habitus

The sociological concept of ‘habitus’ is suited to analyse the food dimensions raised during consultations and how these dimensions are embedded in patients' everyday lives [16, 17, 18]. Habitus is a concept developed by the French sociologist Pierre Bourdieu (1930–2002), who is renowned for his work on understanding how social structures influence people's thinking and actions in everyday life. In short, habitus refers to a set of embodied social structures, such as family and social class, that shape everyday thinking and acting [16, 19, 20].

The following characteristics of habitus are relevant for the current study:

- Social: habitus is fundamentally social; through habitus, individuals internalize and reflect external socio‐cultural structures, such as family and social class. This internalization occurs throughout life ‐ especially in early childhood ‐ rather than being innate.
- Embodied: these socio‐cultural structures shape habits, practices and norms, and are inscribed in the individual's body (rather than in the mind). As a result, individual bodies act in accordance with these ingrained structures. For instance, (class‐based) taste ‐ understood in terms of ‘style’ ‐ is embodied in how individuals act, dress, and eat, often without conscious awareness. Bourdieu highlights how the ways people treat, maintain, feed, and care for their bodies reflect the deepest core of their habitus [21].
- Durable: habitus begins to form in early childhood, which is why earlier experiences play a crucial role in shaping it. This makes habitus durable, long‐lasting, and difficult to change, which is why understanding an individual's history ‐ the temporal aspect of life ‐ is crucial to understanding habitus.
- Non‐cognitive: the most defining characteristic of habitus is based on the characteristics mentioned above: its non‐cognitive, non‐verbal, and non‐reflexive nature [19, 22, 23, 24, 25].

We will use these habitus characteristics as a lens to analyse how food dimensions raised by dietitians and patients during consultations are embedded in patients' everyday lives.

## 2 Methods

This study primarily employs observations, supplemented by informal interviews. Patient‐dietitian interactions were observed during consultations, enabling the direct capture of food dimensions raised. Informal interviews with dietitians provided opportunities to reflect on the consultations and their settings.

### 2.1 Study Setting

In the Netherlands, dietitians are licensed healthcare professionals who have completed a 4‐year bachelor's degree in dietetics. In primary care, they manage a wide range of conditions, including those requiring post‐hospital care. Primary dietetic care is reimbursed for up to 3 h per year under the basic health insurance package, although a compulsory deductible applies. Dietitians visit older patients at home only if the general practitioner (GP) issues a home visit warrant stating that the patient is unable to attend a dietetic practice. Oral nutritional supplements (ONS) are reimbursed if prescribed by a dietitian.

### 2.2 Participants

Participants were dietitians, home‐dwelling older adults and, if present, informal caregivers. The recruitment of dietitians working in primary care in the south‐west of the Netherlands (private‐ and group practices) was done through a general internet search using search terms like ‘dietetics’, ‘malnutrition’ and ‘older adults’ and searches via websites of dietetic geriatric networks and dietetic professional associations. Inclusion criteria were being engaged in the management of malnutrition in home‐dwelling older adults and having minimal 1 year of professional work experience.

Identified dietitians were contacted by email and subsequently by phone. They received both oral and written information regarding all aspects of the study, including its purpose, the observation process (e.g., the researcher not participating in the consultations, sitting at the back of the room, and taking notes), the request to conduct consultations as much as possible in their usual manner, the possibility that the researcher might ask follow‐up questions between consultations, and the estimated time commitment. Dietitians were then asked to permit observations of their consultations with older patients. Older patients who received home‐based dietary consultations were selected by the participating dietitians from their patient files and invited to participate if they met the inclusion criteria: an age above 70 years and diagnosed with malnutrition or at risk of malnutrition. No exclusion criteria were applied. Dietitians cited expected fears of COVID‐19 contamination among older patients as reasons for non‐participation, and also their own time constraints and insufficient numbers of eligible patients.

### 2.3 Data Measurement and Collection

Initial and follow‐up consultations of dietitians with older patients were observed by the first author through home‐visits between November 2022 and March 2023. The first author held 4 pilot observations to get to know situations and interactions that were (im)possible to observe and to become acquainted with consultations and the context in which they take place (e.g. time frame, people's situation at home).

To determine an appropriate sample size, several factors were considered. Sample sizes reported in comparable qualitative observational studies were taken into account, alongside the need to balance depth and richness of data with practical feasibility, and the adoption of an iterative approach to data collection. This allowed emerging insights from ongoing analysis to inform subsequent observations, with the final number guided by the point at which additional data no longer provided meaningful analytical value in relation to the research question. While recognizing that data saturation is a contested concept in qualitative research, it was used here as a pragmatic guiding principle.

An observation guide was developed to structure and focus the field notes, which also included direct quotes. This guide was not used as a strict, all‐decisive format; when deemed necessary, additional field notes were taken. The guide covered general data, like basic characteristics of dietitians (such as age, years of working experience) and patients (such as age, although basic characteristics of patients were only collected when provided by the dietitian), people present during consultation, and more specific data like the wording that was used, and how dietitian and patient attuned to each other's social, cultural and intellectual background. The observation guide was designed to effectively capture food dimensions, making audio recording unnecessary. Recording was also avoided due to frequent caregiver comings and goings, which complicated audio quality and consent. Most importantly, it helped preserve the natural flow of consultations with fragile patients, keeping interactions authentic.

Before and after consultations, informal interviews with dietitians were conducted to reflect on the consultation setting, patient context, reasons for addressing specific issues, and the researcher's influence. Due to their informal nature, interviews were not audio‐recorded, but detailed notes were taken immediately afterward.

### 2.4 Data Analysis

ATLAS.ti 24.2 [26] was used for data analysis. Shortly after each observation, the first author reviewed the field notes, and preliminary findings were used to refine the focus of subsequent observations and inform ongoing sampling decisions. This iterative process continued until data saturation was reached, as additional observations no longer yielded new insights. Contextual data, obtained from the observations and interviews, were used to describe the setting of the consultations.

Two researchers (the first and second author) independently used inductive reasoning to identify food dimensions from the data, guided by the following definition of food dimension: a food dimension is the way in which food integrates in the everyday lives of older adults. This led to a provisional overview of food dimensions. It was also noted who raised a dimension (dietitian, patient, informal caregiver). This overview was then discussed by the first and second author, and differences in interpretations were discussed to increase the quality of analysis. The first and second authors then independently categorized the dimensions into larger clusters. These clusters were also discussed, which led to the final overview of dimension clusters.

Finally, the first and second author assigned the clusters to social, embodied, durable or non‐cognitive characteristics of habitus. We used an intuitive rather than a formal approach as the aim of this clustering was to facilitate practice‐oriented outcomes using habitus as a lens, rather than to develop a conceptual model at a theoretical level.

### 2.5 Ethics and Reflexivity

Ethical clearance was obtained from the Social Sciences Ethics Committee of Wageningen University and Research, approval number 2022‐94. After being informed, both in writing and orally, written informed consent was obtained from dietitians and patients before the consultation. To ensure confidentiality, each participant was assigned a pseudonym.

Observations were conducted by the first author (male), a sociologist and research methods lecturer at a Nutrition and Dietetics Faculty. Data analysis was carried out with the second author (female), a trained dietitian and current researcher/lecturer in innovative nutrition promotion.

The first author sought to minimize influence by staying in the background, limiting eye contact, and responding briefly. He emphasized his role as a researcher focused on everyday practice, not as an expert in dietetic communication or malnutrition management.

## 3 Results

### 3.1 Setting

The study involved the observation of eight dietitians, with a total of 31 observations conducted, after which additional observations no longer yielded new insights [27]. As shown in Table 1, aside from gender, the descriptive characteristics exhibit considerable heterogeneity.

**Table 1:** Descriptive characteristics of dietitians participating in the study.

Dietitian number (patient numbers)	Sex (M/F)	Age	Years experience as dietitian	Years experience in elderly care	Year graduated as dietitian	# Observation hours	# Observed consultation	Employment
1 (1, 2)	f	32	3	1	2020	1	2	Employee
2 (3, 4)	f	32	2	2	2021	1	2	Self‐employed
3 (5–7)	f	60	9	5	2003	1, 5	3	Self‐employed
4 (8–14)	f	39	14	11	2009	7	7	Employee
5 (15–22)	f	35	12	12	2011	6, 5	7	Employee
6 (23–25)	f	22	1	1	2022	2, 5	3	Employee
7 (26–28)	f	27	4	4	2019	3, 5	4	Employee
8 (29–31)	f	32	2	2	2020	3	3	Employee
Totals						26	31	

### 3.2 Setting of Participants and Consultations

Participating dietitians stated in interviews that home visits offer the advantage of allowing for a better understanding of patients' personal, social, and food contexts. However, they also highlighted the challenges, including the demands of travel and the lack of personal space. During consultations, dietitians must balance time constraints, targets – a limited number of home visits are reimbursed by the health insurer – attention to the patient, focus on their needs, and the use of client dossier software (e.g., Intramed, Evry). Sessions typically lasted 60 min initially or 30 min for follow‐ups.

Malnourished older adults often appeared frail, dealing with multiple health issues and largely confined to their homes. The dietitian was never the only healthcare professional involved, and we observed that patients divide their time, energy, and attention among the various professionals in their care network. Patients generally know how to be a patient: they follow the routine; offer the dietitian a seat, and take a step back as she begins the consultation. Patients rarely seem surprised by the topics discussed, as they appear to align with the familiar domain of dietetics. At the same time, patients often carve out space to share their own stories, which are usually related to food but do not necessarily fit neatly into the structure of the consultation.

Informal caregivers, when present, took an active role by asking prepared questions, explaining conversations, showing foods, and encouraging older adults to provide adequate answers. They asked more questions than the patients and engaged closely with the dietitian's advice. Additionally, they challenged their reasons for not following recommendations.

Most patients lived in modest apartment complexes or terraced houses. Most consultations took place in the living room, with the patient seated in a comfortable chair.

### 3.3 Overview of Dimensions of Food

A total of 78 dimensions of food, raised by dietitians, patients and informal caregivers, were identified from the data. These dimensions were grouped into 13 clusters. Table 2 provides an overview of these 13 clusters, starting from dimension clusters that are most frequently raised by dietitians during consultations.

**Table 2:** Clusters of food dimensions raised in consultations by dietitians, patients and informal caregivers. Order based on frequency mentioned by dietitians.

Clusters	Topic
Measuring and knowing	Giving or asking information based on obtained information
Effect of diet therapy	The relation between food intake, gaining weight and (quality of) life
Norms	The health‐related norms that are consciously or unconsciously attached to food
Caring	Food related caring for others, for instance bringing or preparing food
Practical	The financial and physical challenges of obtaining and preparing food and ready‐made meals
Considering	Evaluating options regarding food (e.g. phasing out ONS and increasing intake regular food)
Medical	Food (regular, ONS or via tube) that affects medical conditions like problematic stool, copious mucus, (abdominal) pain and vice versa
Wanting and being able to eat	The will and the possibilities to eat and the lack thereof
Personal preferences	The relation between the person and his/her food choices (regarding to gaining weight)
Structure of the day	Relation between time of the day and (lack of) food intake
Emotion	Emotions related to food; for instance, fear or happiness (enjoyment)
Social	Patient's food choices or regimes in relation to others, for instance partner children or friends
Past and present times	Changes in food choices and preferences over the years

**Figure 1:**
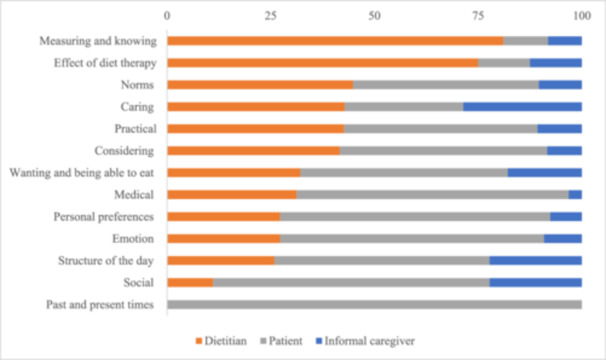
Relative frequency (%) of clustered food dimensions raised during consultations by dietitians, patients and informal caregivers.

Figure 1 illustrates the relative frequency with which food dimensions ‐ clustered into categories ‐ were raised during the consultations by dietitians, patients and informal caregivers. Dimensions within the cluster ‘measuring and knowing’ were almost always raised by dietitians and only rarely by patients or informal caregivers. The same holds for ‘effect of diet therapy’, while ‘past and present times’ is only raised by patients. The other dimensions are raised by both, often including informal caregivers, but with varying frequencies. For example, the ‘social’ dimension is mentioned by dietitians but more frequently by patients and informal caregivers.

### 3.4 Description of Clustered Food Dimensions by Habitus Characteristics

**Figure 2:**
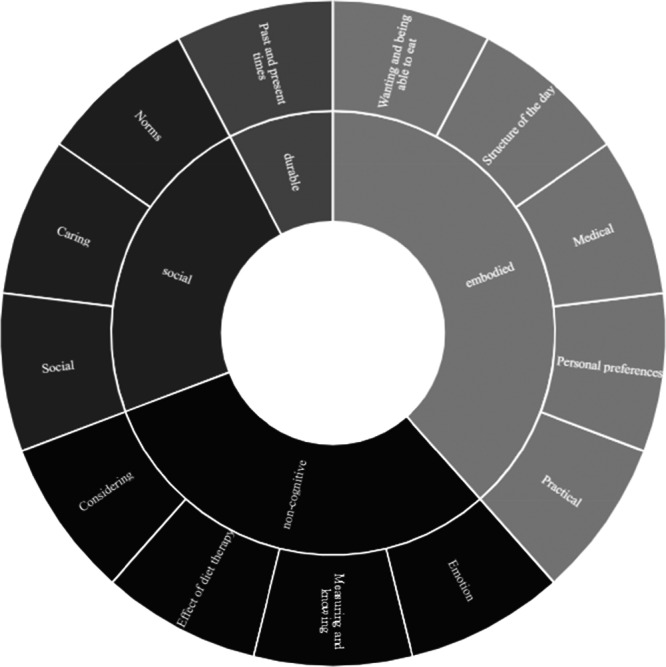
Characteristics of habitus (inner ring) and clustered food dimensions (outer ring).

Figure 2 presents an overview of the dimension clusters categorized by the social, embodied, durable and non‐cognitive habitus characteristics.

What follows is an elaboration of each clustered food dimensions per habitus characteristic.

### 3.5 Social and Clustered Food Dimensions: Norms, Caring and Social

Dietitians referred to and asked about immediate family and friends (dimension cluster ‘social’) but mostly did so in the context of food‐related caring. For instance, a recently widowed patient lost interest in cooking since it no longer felt meaningful to prepare meals just for herself. The dietitian suggested alternatives like dining with neighbors, but she dismissed them as unsuitable (Dietitian8, Patient29 (female)), hereafter referred to as ‘(Dx, Px (gender))’.

Dietitians rarely mentioned broader social influences that shape formative experiences, such as culture or social class. In contrast, patients implicitly reflected these influences through socially acquired and deeply ingrained norms (dimension cluster ‘norms’) of not overeating, even when malnourished. For instance, patients often continued snacking (e.g., on candy or cheese), a behaviour they described as undesirable, implicitly referencing a social norm against excessive snacking. Apart from generally accepted social food norms, patients also referred to more specific norms they picked up in their social environment.

“It is being advised that I should drink cranberry juice.”

(D3, P6 (female))

Patients sometimes also resisted norms when they felt they were imposed on them because of their medical condition.

“That might all be true [dietetic advice], but I'm not going to live like a monk!”

(D6, P23 (male))

Patients often asked dietitians about allowed foods, but dietitians rarely imposed strict rules. Compared to stricter informal caregivers, dietitians were usually the first to reassure patients they were doing well.

In the ‘caring’ dimension cluster, both dietitians and patients involved relatives. Dietitians primarily did so to encourage food‐related care for older adults, while patients mainly shared how they were cared for and expressed their appreciation.

### 3.6 Embodiment and Clustered Food Dimensions: Wanting and Being Able to Eat, Structure of the Day, Medical, Personal Preferences and Practical

In general, dietitians were cautious about suggesting significant changes to food habits (dimension cluster ‘habits’).

“It's not necessary to radically change your food. You're doing fine already. Let's make small changes.”

(D3, P7 (female))

Dietitians provided many practical tips and offered support within the ‘practical’ dimension cluster. e.g., they suggest using a high chair while cooking, explain how to obtain ONS, or discuss which flavors are available or unavailable. Similarly, within the ‘structure of the day’ cluster, dietitians offered practical guidance on maintaining regular mealtimes. e.g., an informal caregiver observes:

“Everything related to eating is different now; one moment the home care service comes, then something else happens, and then something else again. There are no more structured mealtimes.”

(D4, P11 (female))

From a dietetic standpoint, dietitians consistently emphasized the importance of structured mealtimes. When patients mentioned they often forget mealtimes, dietitians offered practical solutions, such as setting an alarm.

Patients often mentioned medical issues, such as pain, medication needs, or bodily sensations like urine odour, as a part of struggles they face in their daily lives. Dietitians responded empathetically.

When the ‘personal preferences’ cluster is considered, it most often relates to food taste. Patients shared their preferences, and dietitians adapted their advice accordingly.

### 3.7 Durability and Clustered Food Dimensions: Past and Present Times

Dietitians seldom discussed food within a historical context (dimension cluster ‘past and present times’). In contrast, older adults did refer to the past in two distinct ways: the topic at hand reminds them of food in the past, e.g.:

“They used to eat rusk with yoghurt.”

(D4, P13 (female))

or to describe a change in what they ate, e.g.:

“I used to like ice‐cream, but not anymore. Last Christmas, I took a little, ew.”

(D4, P14 (female))

“I find it terrible that I can no longer enjoy a glass of wine.”

(D5, P16 (male))

### 3.8 Non‐Cognitive and Non‐Reflexive and Clustered Food Dimensions: Measuring & Knowing, Effect of Diet Therapy, Emotions and Considering

Dietitians spent a considerable amount of consultation time on taking a patient's dietary history (dimension cluster ‘measuring and knowing’). In doing so, they relied on instrumental or means‐end rationality where the means is to get to know a patient's food intake and the end is to give dietary advice based on intake. We observed a two‐fold discrepancy between dietitians' and patients' reasoning.

Instead of means‐end‐rationality, patients often engaged in associative reasoning, which led them to diverge from dietary history taking and talk about food in a broader, more experiential context. This included various dimensions of food in their lives, such as emotions (dimension cluster ‘emotions’) or taste (dimension cluster ‘personal preferences’). For example, one patient, who could only eat small amounts of regular food due to tube feeding, described a dish as “heavenly” (D4, P8 (male)). Another emphasized the importance of buying high‐quality fish when having guests over (D8, P31 (male)). Similarly, an informal caregiver sought dietary advice that required minimal cooking, as he was unable to cook (D5, P20 (female)). We often experienced patients' strong urge to talk about these aspects of food. Their desire to do so is so profound that it disrupts both the structure of the consultation, which is primarily centered on taking dietary histories, and the instrumental mode of reasoning applied by dietitians.

Secondly, food‐related rational reasoning appears to be challenging for patients. For instance, one patient could passionately discuss Spinoza's abstract philosophy yet still relied on his dietitian and son for guidance on consuming additional snacks (D8, P31 (male)). We also see that informal caregivers often stepped in to clarify and supplement patients' responses. Patients consistently attempted to engage with dietary recommendations but did so in ways deeply rooted in their personal experiences and daily lives. e.g., when a dietitian advised a patient to consume extra butter, the patient responded:

“I do not promise I will take extra butter.”

(D5, P21 (female))

While adding extra butter may seem like a straightforward and rational choice, this was not necessarily the case in the everyday lives of older adults. The same holds for the dimension cluster ‘effect of diet therapy’. Dietitians inquired about the effects of the implemented dietary changes, but patients found that hard to answer.

The ‘considering’ dimension cluster refers to patients weighing different food (and medicine or ONS) options. This occurs, for example, when medication interacts with food, and dietitians helped patients make informed choices. ‘Considering’ is therefore closely tied to daily life, with little abstract reasoning involved. When rational considerations arise, patients often struggled. For instance, a patient feared cooking due to salt restrictions rather than logically assessing suitable ingredients (D5, P18 (male)).

## 4 Discussion

This study examined the dimensions of food that were raised in consultations between dietitians and home‐dwelling older adults. 78 dimensions were identified and clustered into 13 categories. While most dimensions were raised by both dietitians, patients, and informal caregivers, significant differences were observed. Dietitians primarily focused on measurable dimensions of food, like intake and improvement, although they did not limit themselves to these areas. In contrast, patients placed greater emphasis on the emotional, social, and temporal dimensions of food.

Habitus refers to a set of embodied social structures, such as family and social class, that shape thinking and acting in everyday life [19]. When applied to the observed consultations, we consistently see that food dimensions raised by dietitians were less aligned with habitus characteristics than the food dimensions raised by patients. Dietitians do raise social and durable aspects, but both serve as functional tools to address immediate malnutrition issues rather than the broader social and temporal context of food in patients' lives. Dietitians also consider the embodied aspect of habitus by suggesting small, manageable changes and by offering practical tips like food preparation advice, allowing patients to maintain existing habits. Dietitians primarily engage patients' rational and cognitive faculties to discuss food in their lives with the aim to uncover the effects of diet therapy. This contrasts with the non‐cognitive nature of habitus, an intuitive, non‐reflexive mode of engaging with food that becomes evident, e.g., in the emotional responses elicited during discussions about food.

In line with Bourdieu's theory of habitus, our observations show that patients often rely on associative, everyday reasoning and display limited cognitive reflection on their food‐related behaviors. This contrast in reasoning between patients and dietitians affects both the dietary information sought and general communication about food.

We also found that patients ask few diet‐related questions and often appear passive during consultations. The evident mismatch between what patients and dietitians emphasize, and between the assumed patient agency and the observed lack thereof, supports the relevance of applying the habitus‐concept in this context.

Our study reveals subtle yet significant differences in how food is discussed during consultations; differences that can influence both communication effectiveness and patient adherence. Patients are best understood not only as autonomous individuals, but also as people embedded in social, historical, and class contexts. To better align with patients' habitus, dietitians could enrich a rational, functional approach by involving patients' social environments, eating histories, tastes, and everyday practices. Being aware of these aspects, including how someone decorates or arranges their home, can reveal the values that shape dietary behaviour.

In dietetics education, curricula could invite students to consider a habitus‐sensitive perspective rooted in Bourdieu's thinking. Alongside behavioural change models [28], students might be encouraged to view their future clients' choices, lifestyles, and home settings as emerging from broader social and cultural contexts. Providing opportunities for prolonged experiential learning, such as observing, interviewing, or visiting patients' homes over an extended period, can help students appreciate how social patterns and lived experience matter for nutritional care. Education should also facilitate students' in‐depth reflection on the demands imposed by biomedical discourses, efficiency‐driven practices, patient‐centered care, and their own habitus [29].

This study uniquely focuses on consultations in malnutrition management, emphasizing the role of food dimensions in everyday life. In contrast, most studies take a broader, interview‐based approach covering multidisciplinary cooperation, dietitian‐patient rapport, and aspects of person‐centred care (PCC) and shared decision‐making [30, 31].

Dietitians' focus on the ‘measuring and knowing’ dimension is consistent with findings from Suarez et al. [32] Our interpretation that dietitians often act on basis of functional rationality is in line with Palermo, Reidlinger and Rees [33]. From the patient's perspective, Nyberg et al. [22], found that older adults engage rationally with food, seemingly contrasting the habitus framework and our study. This discrepancy likely reflects their focus on older adults with motor difficulties adapting eating behaviors in social contexts.

We found that dietitians engage in person‐centered care (PCC) by responding to individual patient preferences, needs, and values, which is in line with studies examining dietitian perspectives [5]. Dietitians avoid authoritarian approaches, build positive relationships, and offer tailored advice, but often treat social, cultural, and temporal dimensions superficially and primarily as functional to treatment goals, possibly due to external efficiency demands [5, 34]. Calls for PCC in dietetics [4, 35, 36] are important, yet practical implications are still lacking when it comes to addressing patients' food behaviours and attitudes and embedding these into their everyday lives, particularly given the multidimensionality of food. Our findings show that using the lens of habitus can enrich and complement PCC by bringing in Bourdieu's thinking about the deep connection between the food attitudes and behaviors and habitus. PCC in dietetics should emphasize the social and temporal dimensions of food practices over cognitive and reflective aspects, to better align consultations with patients' everyday thinking and acting.

### 4.1 Strengths and Limitations

A strength of this study is that observations were used to examine interactions in everyday consultation contexts between dietitians, patients, and, occasionally, informal caregivers. Unlike studies focusing on patients outside the consultation context [7, 37], on healthcare providers [38, 39, 40], or on both [7], this approach enables direct insights into consultations without reliance on retrospective reflections, hence avoiding biases.

While our study had strengths, it also had limitations. Dietitian interviews provided context, but interviewing patients and caregivers would have offered deeper insight into their motivations. This will be addressed in a follow‐up study. The geographical focus on the southwestern Netherlands may limit generalizability to other regions or countries. However, since dietitians worldwide treat malnutrition in home‐dwelling older adults and face similar curricular and efficiency demands, our findings remain relevant internationally, especially as patients everywhere hold food‐related attitudes and behaviors, though shaped by differing habitus.

## 5 Conclusions

We conclude that dietitians and patients focus on different dimensions of food during consultations. Dietitians tend to emphasize rational and functional dimensions, whereas patients highlight social, emotional, and temporal dimensions. Our application of Bourdieu's habitus concept suggests that dietitians' advice, while patient‐centered, is often shaped primarily by pragmatic considerations and by an emphasis on the individual patient. This focus can make it more difficult to account for the historical and social aspects of food in patients' lives, meaning that advice is not always fully embedded in their everyday practices and thus may not consistently connect with patients' food attitudes and behaviors.

## Author Contributions

Matthijs Fleurke designed the study and conducted the observations and interviews, and together with Laura Bouwman, performed the data analysis. Matthijs Fleurke, Laura Bouwman, Jacqueline Langius and Spencer Moore contributed to the conceptualization, writing and editing of the manuscript.

## Ethics Statement

Approval was obtained from the Wageningen University & Research Research Ethics Committee: approval number 2022‐94.

## Conflicts of Interest

The authors declare no conflicts of interest.

## Data Availability

The data that support the findings of this study are available on request from the corresponding author. The data are not publicly available due to privacy or ethical restrictions.
